# S176. SYSTEMATIC REVIEW AND META-ANALYSIS OF MAGNETIC RESONANCE IMAGING FINDINGS IN 22Q11.2 DELETION SYNDROME

**DOI:** 10.1093/schbul/sby018.963

**Published:** 2018-04-01

**Authors:** Guido Maria Lattanzi, Cristina Scarpazza, Fabio Di Fabio, Philip McGuire, Giuseppe Sartori, Simon B Eickhoff, Stefania Tognin

**Affiliations:** 1 Institute of Psychiatry, Psychology & Neuroscience, King’s College London; 2 Institute of Psychiatry, Psychology & Neuroscience, King’s College London, University of Padua; 3 Sapienza University of Rome; 4 University of Padua; 5 Institute for Systems Neuroscience Heinrich-Heine University Düsseldorf

## Abstract

**Background:**

Since the 22q11.2 Deletion Syndrome (22q11.2 DS) is the most important genetic model for psychotic disorders, an increasing interest in its brain structural and functional abnormalities has arisen in the last decade. However, studies so far have reported inconsistent findings. Therefore, the aims of the present study are 1) to systematic review the literature on structural and functional brain abnormalities associated to 22q11.2 DS and 2) to identify the most consistently reported abnormalities through a meta-analysis of structural (sMRI) and functional magnetic resonance imaging (fMRI) studies.

**Methods:**

The following electronic databases were systematically searched: PubMed, ETHOS, Kings Open Portal, EMBASE, MEDLINE, PsycINFO and CINHAL. Studies were included if they presented original data, were written in English, had a sample size larger than 5, had a healthy control comparison group, and if they reported results from a whole brain analysis. As we were interested in identifying abnormalities in both brain structure and function, in the systematic review we included studies that used different imaging techniques (i.e. sMRI, fMRI and diffusion tensor imaging (DTI)). The meta-analysis was performed with studies reporting results in standardised-space coordinates (e.g. Talairach), using the Activation Likelihood Estimation (ALE) method. Results were corrected at cluster level with family wise error correction (p=0.01) and 1000 permutations.

**Results:**

Seventy-three original articles were included in the systematic review, 25 of these were also included in the meta-analysis. Forty-two sMRI, 23 fMRI and 11 DTI articles were retrieved. Only one study performed a direct comparison between 22q11.2 DS individuals with and without psychosis.

The systematic review revealed that the most affected areas were the frontal middle gyri bilaterally, the posterior cingulum bilaterally, the right cuneus, the precuneus bilaterally, the right superior temporal gyrus, the left parietal inferior gyrus and the left side of the cerebellum.

The meta-analysis revealed consistent abnormalities in a cluster located in the inferior parietal lobe (4936 voxels, peak of activation in the coordinate -44 -52 48) and extending to the superior temporal gyrus, supramarginal gyrus and precuneus. A second cluster of consistent activation is found in the posterior cingulate cortex (3104 voxels, peak of activation in the coordinate 6 -50 16).

**Discussion:**

The systematic review revealed widespread abnormalities throughout the brain, mainly within areas involved in visual and speech processing, language, and within association areas. The meta-analysis of structural and functional studies revealed consistent abnormalities in the inferior parietal lobe, an area consistently found affected in psychosis.

Only few studies on 22q11.2 DS individuals with psychosis were available and most studies included young individuals (mean age 15.12) rather than adults. 22q11.2 DS is one of the most compelling genetic models of schizophrenia, however most imaging studies do not provide clinical data on psychotic symptoms. This could partially be explained by the relatively low mean age of the overall sample; some participants could have been too young to manifest psychotic symptoms. Finally, the present study does not allow to make inferences on brain changes overtime as longitudinal studies were scarce.

Future studies should adopt a longitudinal design and investigate brain abnormalities in adults with 22q11.2 DS displaying symptoms of psychosis. This would help to clarify the brain structural and functional features associated with this particular form of psychosis and their longitudinal course.

